# Letter to the Editor “Meta-analysis of longitudinal comparison of transcatheter versus surgical aortic valve replacement in patients at low to intermediate surgical risk”

**DOI:** 10.1097/JS9.0000000000002659

**Published:** 2025-06-05

**Authors:** Neda Roshanravan, Faezeh Tarighat, Rezayat Parvizi

**Affiliations:** Cardiovascular Research Center, Tabriz University of Medical Sciences, Tabriz, Iran

*Dear Editor*,

We want to thank the authors of “Meta-analysis of longitudinal comparison of transcatheter versus surgical aortic valve replacement in patients at low to intermediate surgical risk” for their thorough discussion of how the application of transcatheter aortic valve replacement (TAVR) and surgical aortic valve replacement (SAVR) can impact patients’ longevity^[1]^. This provides valuable insights into the short- and long-term outcomes of SAVR and TAVR. However, this investigation still has certain limitations that require further explanation and discussion.

Conduction abnormalities following both techniques, a rare but noteworthy complication, pose a burden on patients by lowering the quality of life and survival rate and prolonging hospital stays. Empirical data indicate that permanent pacemaker implantation (PPMI), as an important disadvantage of aortic valve replacement (AVR), is independently correlated with diminished long-term survival^[2]^. These effects are more prominent in younger patients with higher life expectancies, as younger patients are more likely to suffer long-term complications. New techniques, such as minimally invasive aortic valve replacement (MIAVR), sutureless implant aortic valve replacement, and TAVR, have shown improved outcomes through reduced perioperative bleeding, shorter cross-clamp time, and lower rates of acute kidney injury. Unfortunately, they are accompanied by higher (TAVR) or similar (MIAVR) incidences of PPMI in comparison to standard SAVR^[3]^. Nowadays, a crucial clinical challenge in structural heart interventions is developing novel aortic valve replacement techniques that minimize injury to the cardiac conduction system, thereby reducing postoperative PPMI. The Ahmed research group documented a significantly increased risk of PPMI at 30 days (RR: 2.62, 95% CI: 1.40–4.91), 1 year (RR: 2.19, 95% CI: 1.24–3.87), and 2 years (RR: 2.74, 95% CI: 1.31–5.71) following TAVR compared to SAVR. Other preliminary studies have reported PPMI incidences from 2.7% to 5% after SAVR^[2,3]^. Interestingly, based on recent quantitative analysis, the novel modified technique reduced the rate of PPMI to 0.8% (95% CI, *n* = 3/183) by attenuating the occurrence of complete heart block (*P* = 0.024)^[4]^. This new surgical technique involves 2–3 felted mattress sutures from the outer side of the aortic valve in the commissure between the right and non-coronary cusps. This adjustment reduces the likelihood of damage to the conduction fibers adjacent to the valve. Based on these findings, the “new” SAVR patients experienced fewer instances of complete heart block postoperatively. Furthermore, the new technique demonstrated a 1.2 percentage point reduction in mortality (2.5% vs. 3.7%; absolute risk reduction of 1.2%) compared with the standard SAVR procedure.

**HIGHLIGHTS**

- Emphasis on PPMI as a critical limitation in valve replacement techniques.
- Introduction of a Novel SAVR technique with significantly reduced PPMI rates.
- Comparative Risk data supporting the need for innovation.

In summary, this new technique can lead to a measurable reduction in PPM need after AVR and thereby improve the patient outcome and enhance quality of life. Ultimately, it will reduce healthcare costs by minimizing complications related to PPMI.

We recommend considering this technique, particularly in patients with a higher preoperative risk of requiring a permanent pacemaker after surgery. Further studies are needed to assess its potential benefits and drawbacks.

This article has not utilized artificial intelligence (AI) tools for research and manuscript development, as per the TITAN Guideline^[5]^.

## Data Availability

Not applicable.
